# Survey data on consumer circular economy behaviours: Focus on repair, reuse, recycling, refurbishment, and sharing in electronics and furniture across Germany, Italy, India and South Africa

**DOI:** 10.1016/j.dib.2026.112885

**Published:** 2026-05-22

**Authors:** Altamash Bashir, Lena Senft, Christian A. Klöckner

**Affiliations:** Norwegian University of Science and Technology, Department of Psychology, 7491 Trondheim Norway

**Keywords:** Circular economy, Citizen behaviour, Electronics, Furniture

## Abstract

This dataset reports individual-level survey data on consumer attitudes and behaviours toward the circular economy (CE) in the electronics and furniture sectors across Germany, Italy, India, and South Africa (N = 4,216; at least 1,000 respondents in each country; March–April 2024). It covers demographics, product ownership, purchasing patterns, awareness, sustainable consumption habits, willingness to pay for circular products, and perceived motivations and barriers. The study focuses on five CE practices—repair, reuse, recycling, refurbishment, and sharing—and provides country-level descriptive statistics. The dataset, accompanying codebook, and survey instrument enable cross-cultural comparisons and support methods ranging from regression and structural equation models to scenario analyses and feeding Agent-Based Models as well as informing material flow models. These data can be used to inform research, policy, and business strategies that promote CE adoption.

Specifications Table

**Table:** 

Subject	Social Sciences
Specific subject area	Circular economy practices in the electronics and furniture sectors
Type of data	Raw (uncoded) survey responses (Excel format) and recoded survey responses (available in CSV, SPSS, Stata, and R formats), accompanied by a codebook (Excel format) and the survey instrument (PDF)
Data collection	Data were collected through the Efficience3 online panel in March–April 2024 across four countries (Germany, Italy, India, and South Africa). Respondents were adults (≥18 years) recruited via stratified online sampling. The data were delivered in raw format and processed in R for analysis. Raw (uncoded) and recoded individual-level responses, an accompanying codebook, and the survey instrument.
Data source location	This is a cross-sectional online survey conducted in March–April 2024 with 4,216 adult respondents (≥18 years) from Germany, Italy, India, and South Africa, with a minimum of 1,000 participants per country.
Data accessibility	Repository name: Zenodo.org Data identification number: https://doi.org/10.5281/zenodo.17641408 Direct URL to data: https://doi.org/10.5281/zenodo.17641408
Related research article	A research article [1] based on this survey has been submitted to a journal and is currently under review.

## 1 Value of the Data

• The dataset provides unique survey data from Germany, India, Italy, and South Africa on consumer attitudes and behaviours toward circular economy (CE) practices in the electronics and furniture sectors, enabling cross-cultural comparisons.
• The data can be used for correlational and regression-based analyses, as for example structural equation modelling (SEM), to examine how awareness, willingness to pay, and perceived social, financial, and infrastructural barriers influence consumer engagement in CE practices.
• The dataset can be applied in country or subgroup comparison to analyze subgroup specific drivers and barriers associated with consumer engagement in CE practices (e.g., from intention to adoption of repair, reuse, recycling, refurbishment, and sharing).
• The data allows studies of spillover effects across CE practices, for example whether engagement in recycling is associated with greater likelihood of repair, reuse, refurbishment, or sharing.
• Based on the identified drivers and barriers, the dataset can inform policy, education, and business strategies, supporting tailored programs, outreach campaigns, and product/service development.
• The data provides a basis for scenario building and informs advanced models (including agent-based models, ABMs, economic models, and material flow models) to simulate consumer behaviour under different policy scenarios. They can also support research on strategies to enhance resilience of sustainable consumption systems.

## 2 Background

Consumer behavior plays a crucial role in supporting CE practices. Many studies have highlighted the importance of understanding consumer engagement in these practices for facilitating the transition toward more sustainable consumption patterns [[2], [3]]. However, existing research has often been limited to single countries, single sectors, or selected behaviours, making it difficult to compare consumer engagement in CE practices across contexts. In addition, the role of consumer behaviour is still relatively underexplored or coarsely explored [2,4,5]. The motivation for the present survey was therefore to generate cross-country data on the main dimensions of consumer engagement in CE practices across countries and product domains. These dimensions include awareness and familiarity with CE concepts, intentions toward implementing CE practices, motivations, barriers, willingness to pay or invest in circular product features, and consumer engagement in CE practices such as repair, reuse, recycling, buying refurbished products, and sharing. Motivation refers to the perceived reasons such as environmental concern, expected economic benefit, and social influence that may encourage consumers to engage in CE practices. Barriers refer to factors such as cost, convenience, and the availability or quality of circular products. Survey-based research has been widely used to investigate consumers’ attitudes and behaviors related to CE practices and sustainable consumption (Kirchherr et al., 2018; [6,7]). Building on this body of literature, the present survey provides the first large, cross-country dataset on consumer engagement in CE practices, including awareness and understanding of central CE principles covering four countries - Germany, Italy, India, and South Africa - and two product domains: electronics and furniture. This scope and scale are distinctive, as prior evidence has largely been limited to single-country or single-sector analyses. The survey concentrates on domains under-represented in CE research and national statistics (electronics, furniture) and captures participation in five comparatively less-studied practices: repair, reuse, recycling, refurbishment, and sharing. The survey for this study was developed following an extensive literature review of consumer behaviour and engagement in the context of CE practices across product domains and regions. Based on this literature review the survey questionnaire was constructed specifically for this study and included several adjusted questions following examples from the literature and ad-hoc questions to assess respondents’ engagement within CE practices.

The dataset serves two main purposes:

• At the macro level, it provides consumer-side data that can be integrated into global climate and resource models. Existing models rarely account for material stocks and circular flows such as repair, reuse, recycling, refurbishment, or sharing. By incorporating citizen data on these practices, the dataset enables more accurate assessment of the mitigation potential of CE strategies.
• At the micro level, the survey captures consumer awareness, motivations, perceived barriers, and lifestyle choices related to CE. This makes it possible to analyze factors shaping attitudes and behaviours, and to compare differences across cultural and geographic contexts.

**Fig. 1:**
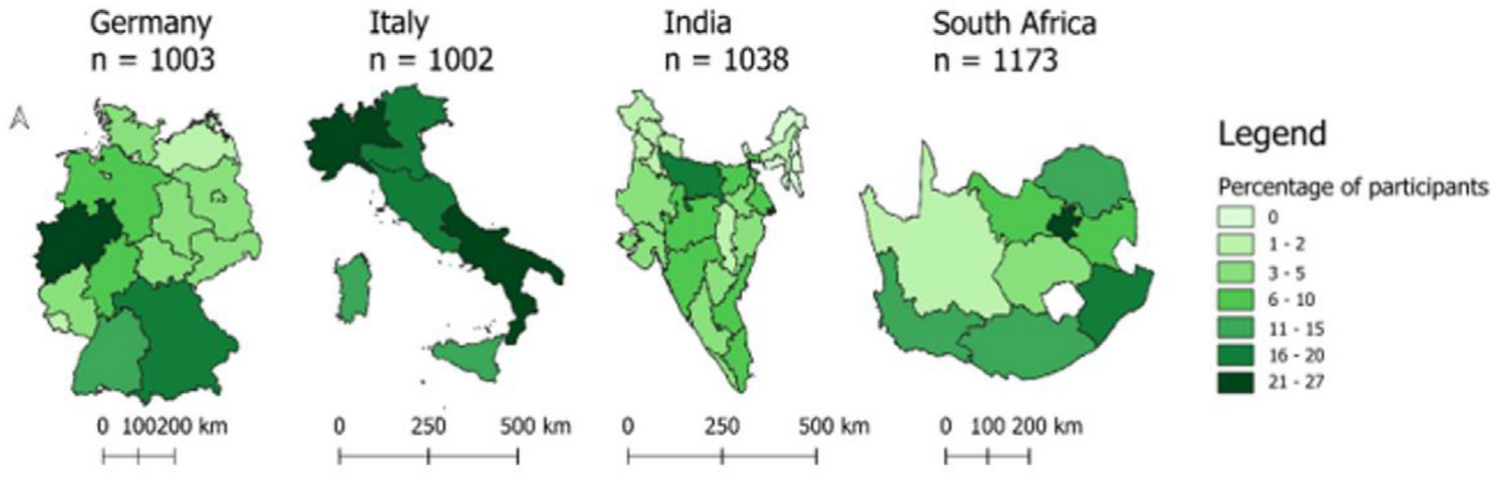
Percentage of study participants per administrative region in Germany, Italy, India, and South Africa. *Note*. Choropleth maps display the percentage of participants per administrative region using a consistent seven-class color scale (Natural Breaks, Jenks). Maps are shown at different scales to ensure visual comparability across countries. Data are projected in ETRS89 / UTM Zone 32N (EPSG:25832) for Germany and Italy, WGS84 / UTM Zone 44N (EPSG:32644) for India, and WGS84 / UTM Zone 35S (EPSG:32735) for South Africa. Administrative boundaries sourced from Eurostat (NUTS 2021) for Germany and Italy, GADM (version 4.0) for India, and OCHA COD-AB (2020) for South Africa.

Together, these contributions address critical gaps in both global modelling and behavioural research. The sample comprised N = 4,216 respondents—Germany (n = 1,003), Italy (n = 1,002), India (n = 1,038), and South Africa (n = 1,173)—and provides broad demographic and geographic coverage across major states and provinces in each country. Fig. 1 shows the geographic distribution of the survey participants across the four countries.

## 3 Data Description

This dataset provides large scale cross-country survey data on consumer behaviour related to CE practices in the electronics and furniture sectors.

The dataset is structured into six thematic sections covering both socio-demographic background and CE-related behaviours:

• Demographics and socioeconomic factors.
• Awareness and familiarity with CE.
• Product ownership and consumption patterns.
• Adoption of CE practices.
• Willingness to pay for CE products and features.
• Intentions, barriers, social influence and motivations for CE practices.

To provide a comprehensive overview, descriptive statistics are reported in Table 1, Table 2, Table 3, Table 4, Table 5, Table 6, covering demographics, household composition, and material possessions across the four countries. These descriptive summaries allow researchers to quickly assess sample composition and the availability of variables for secondary analysis.

**Table 1:** Sources of national population statistics used by Efficience^3^ to set sampling quotas (by country).

	Country			
Variable	Germany	Italy	India	South Africa
Region	Eurostat. (2024, February 1). *Population on 1 January by age, sex and NUTS 2 region – Extract from 01/02/2024* [Data set]. European Union. https://data.europa.eu/data/datasets/cm6tepfivioxoiebggok6q?locale=en	Eurostat. (2024, February 1). *Population on 1 January by age, sex and NUTS 2 region* [Data set]. European Union. https://data.europa.eu/data/datasets/cm6tepfivioxoiebggok6q?locale=en	United Nations, Department of Economic and Social Affairs, Population Division. (n.d.). *World Population Prospects* [Data tool]. https://population.un.org/wpp/	Bureau of Market Research. (2021, July 16). *Mid-2021 population estimates for South Africa by province, district and local municipality*. https://bmr.co.za/2021/07/16/mid-2021-population-estimates-for-south-africa-by-province-district-and-local-municipality/
Age				StatBase. (n.d.). *Population by age groups – South Africa* [Data set]. https://statbase.org/data/zaf-population-by-age-groups/
Gender			Statistics Times. (n.d.). *India sex ratio 2023* [Webpage]. https://statisticstimes.com/demographics/country/india-sex-ratio.php	Statistics South Africa. (n.d.). *Census 2022 data portal* [Data tool]. https://census.statssa.gov.za/#/
Education	Statistisches Bundesamt (Destatis). (n.d.). *Educational attainment of the population in Germany*. https://www.destatis.de/EN/Themes/Society-Environment/Education-Research-Culture/Educational-Level/Tables/educational-attainment-population-germany.html	Organisation for Economic Co-operation and Development (OECD). (n.d.). *OECD Education GPS – Indicator explorer* [Data tool]. https://gpseducation.oecd.org/IndicatorExplorer		
Income	Statista. (2024). *Household net income per month* [Data set]. https://www.statista.com/statistics/263265/net-monthly-income-of-private-households-in-germany/	Ministero dell’Economia e delle Finanze (MEF). (2023, May 3). *Statistiche sulle dichiarazioni dei redditi persone fisiche Irpef e dichiarazioni IVA – Anno di imposta 2021*. https://www.finanze.gov.it/it/archivi/notizie/dettaglio-notizie/Statistiche-sulle-dichiarazioni-dei-redditi-persone-fisiche-Irpef-e-dichiarazioni-IVA-anno-di-imposta-2021/	Statista. (n.d.). *Average monthly household income in India from 2016 to 2021* [Data set]. https://www.statista.com/statistics/653897/average-monthly-household-income-india/	No source reported by the external survey provider

*Note.* The sources listed were compiled by Efficience^3^ and used to define quota targets; the authors did not independently verify the underlying population estimates.

In the following section, the six aforementioned thematic sections are described in terms of their specific items and measurement scales.

### 3.1 Answers and measures of survey data

The dataset includes demographic, socioeconomic, behavioural, and psychological variables, measured using a mix of categorical, ordinal, binary, and continuous formats. The subsections below describe how these variables were captured and coded.

### 3.2 Demographics and socioeconomic factors

Age was reported as a continuous variable. Gender was coded categorically (male, female, other). Education was measured with country specific ordinal categories, while household income was reported in income brackets (ordinal) adjusted to each country. Occupation was captured as a categorical variable (e.g., full time, part time, student, unemployed, retired). Residence type (e.g., single-storey house, apartment) and location were coded using country specific categories distinguishing metropolitan/urban/city, town/suburb, and rural areas. Region/state was captured as categorical variable by country (specifically, German Bundesländer, Italian macro regions, Indian states/UTs, South African provinces).

### 3.3 Product ownership and consumption patterns

Respondents reported the number of furniture and electronic items owned using categorical ranges (e.g., none, 1-2, 3-4; with extended ranges up to “more than 20” for some items). Some variables require numerical entries such as percentages (e.g., share of products repaired or reused) or counts (e.g., number of devices owned).

### 3.4 Awareness and familiarity with CE

Familiarity with CE concepts and with recycling systems for electronics or furniture was measured on five point Likert scales (1 = not at all familiar, 5 = very familiar). Awareness of central CE principles and sustainability topics was captured using similar five point scales.

### 3.5 Attitudes, barriers, and motivations

Attitudinal and psychological measures were operationalized using Likert type scales tailored to each construct, including:

• Agreement (1 = strongly disagree to 5 = strongly agree),
• Perceived effectiveness (1 = not at all effective to 5 = very effective),
• Perceived influence (1 = no influence to 5 = very strong influence), and.
• Likelihood (1 = very unlikely to 5 = very likely).
• Barriers (e.g., cost, availability/quality of CE options, convenience) and motivations (e.g., environmental concern, economic benefit, social image) were assessed on five point scales.

### 3.6 Adoption of CE practices

Actual behaviours and behavioural frequency (e.g., reuse, repair, recycling) were measured on four-point scales (e.g., never, rarely, sometimes, always), including items on the extent of repair and similar degrees of engagement.

### 3.7 Willingness to invest and pay

Willingness to invest in circular product features (e.g., repairability) was measured on four-point scales, while willingness to pay extra was reported as a numeric percentage (0-100%).

### 3.8 Coding of variables

• Continuous: age; numerical entries (percentages, counts).
• Categorical: gender, residence type, location categories, occupation, region/state; ownership ranges.
• Ordinal: education, income brackets; frequency/extent scales.
• Binary: dichotomous future consideration items (e.g., “would consider doing it more” vs. “would not consider”).
• Likert scales: five point (agreement, familiarity, effectiveness, influence, likelihood) and four point (frequency, extent, willingness).

This combination of categorical, ordinal, binary, and continuous variables allows both descriptive analyses and advanced statistical modelling of consumer behaviour in the context of CE practices.

## 4 Experimental Design, Materials and Methods

The survey was developed following an extensive literature review of consumer behaviour in the context of CE practices across product domains and regions. The selected dimensions were included to capture the most important aspects of consumer engagement in CE practices across countries and product domains. These dimensions were identified in the literature review and included awareness and familiarity with CE concepts, product ownership and consumption patterns, consumer engagement in repair, reuse, recycling, buying refurbished, and sharing, willingness to invest in or pay for circular product features, and intentions, social influence, motivations, and barriers. The questions were informed but not derived from previously validated survey questions or scales. Instead, the questionnaire was developed specifically for this study. A short internal survey among project partners, including teams working with material flow models, provided additional feedback and guided the selection of countries, product categories, and CE actions to be included. The feedback from the project partners focused mainly on the selection of countries, and product categories, ownership and consumption pattern. The inclusion of these variables was useful for later modelling, projection and comparative analysis. The partner members also emphasized keeping the questionnaire understandable for general consumers rather than expert audiences. The survey was pilot-tested with a small group of 10 respondents from each country to identify potential ambiguities and ensure that the questions were easily understood. Minor revisions were made based on pilot feedback to improve wording and item clarity before full implementation.

The questionnaire was originally developed in English and subsequently translated into the respective national languages. The authors, who are native speakers of two of the languages, translated the survey into Hindi and German. Another member of the research group translated the survey into Italian. The questionnaire was translated into Zulu by a professional translation agency approved by the university. Additionally, the company responsible for administering the survey used its professional translators to review and refine the translations to ensure accuracy, readability, and consistency. To further ensure clarity and reliability, a pilot test was conducted with 10 participants in each country. Based on the feedback received, minor revisions were made before the full implementation of the survey.The survey was administered by the company, which handled online recruitment, implementation, and quality control. Standard quality-control procedures (e.g., bot detection and verification checks) were applied to ensure respondent authenticity and data reliability. No additional handling of missing or inconsistent responses was undertaken by the research team; the fieldwork provider delivered a cleaned dataset after applying its standard data-processing pipeline. During fieldwork, quality screening targeted speeders, straight liners, implausible values, and duplicates. Interviews meeting these criteria were removed, with manual review where an interview triggered only a single rule. The screening rules comprised:

• Birth year implausible (< 1920).
• Q47 (expected additional years of product life through reuse/repair): extreme values (> 35 years).
• Straight lining on Q62 (barriers) and Q63 (motivations); if only one of the two was straight lined, the case was retained after review.
• Pattern flag: Q26 (engagement in practices) coded 1 for all items and Q62 item 1 or 2 for all modalities.
• Q30 (number of furniture items): flagged for case-by-case review considering dwelling size and household size.
• Q48 (action after failed self-repair of electronics; “other, specify”): text responses reviewed for validity.
• Interview duration < 7minutes.
• Duplicate IP addresses.

As a result, the provider removed interviews for quality reasons: Germany (n = 359), Italy (n = 440), India (n = 2,927), South Africa (n = 327); total n = 4,053.

In total, 8,269 interviews were completed across Germany, Italy, India, and South Africa between March and April 2024. After providing quality control and exclusions (n = 4,053), the analytic sample comprises N = 4,216 adults (≥18 years; ≥1,000 per country). Stratified quota sampling was implemented to align the sample with national distributions of gender, age, education, income, and region. Quota targets were derived from national population statistics compiled by Efficience3 (see Table 1 for source details). For education, quotas were set for Primary school, Secondary or vocational school, Bachelor, and Master and above, while the No education category was not quota-tracked (to avoid exclusions) but retained and later adjusted via weighting. Quotas were targeted within ±2% initially; during fieldwork, tolerances were widened for income (to ±5% in Germany and ±6% in Italy), and education (to ±4% in Italy and ±8% in South Africa and India) to maintain recruitment progress. The provider supplied survey weights (variable “weight”) jointly adjusting for these five variables; no trimming/winsorization was applied by the provider. For analysis, we used the provider weights and rescaled them to mean 1.00 (no trimming). Author-computed diagnostics on the rescaled weights indicate modest dispersion (CV ≈ 0.21; 90% of weights between 0.65 and 1.33) and a small design effect (D_e_ff ≈ 1.04); see Table 2 for full diagnostics of the sampling weights. National target margins used for stratification/weighting are listed in Table 3, and country-specific alignment of the achieved samples to these targets is presented in Table 4, Table 5, Table 6, Table 7. Descriptive results are reported both unweighted and weighted (see Tables 8,9,10–13), where unweighted statistics reflect the raw composition and weighted statistics align estimates with national population parameters. This dual reporting allows future users to select the version most appropriate to their analytical needs.

**Table 2:** Diagnostics of Sampling Weights (Variable: weight)

**Metric**	**Value**
Sample size, *N*	4,216
Sum of weights, *N*	4,216.0
Mean, *M*	1.00
Standard deviation, *SD*	0.208
Coefficient of variation, *CV*	0.208
Minimum / Maximum	0.491 / 2.155
Median (p50)	1.003
80% interval (p10–p90)	0.702 – 1.227
90% interval (p05–p95)	0.646 – 1.325
Interquartile range (IQR)	0.244
Share of weights > 4	0%
Effective sample size, *n_eff_*	4,040.9
Design effect (weighting), *D_eff_*	1.043
Mean prior to rescaling	0.949

*Note.* Weights were rescaled to *M* = 1.00 (mean normalised); no trimming was applied. *CV* = *SD* / *M. n_eff_* = (Σ*w*)^2 / Σ*w*^2. *D_eff_* = *N* / *n_eff_*. Percentiles refer to the distribution of the rescaled weights. Threshold for ‘large’ weights: *w* > 4.

**Table 3:** National target distributions used for stratification and weighting (by country).

		Country			
Variable	Category	Germany	Italy	India	South Africa
		*n* = 1003	*n* = 1002	*n* = 1038	*n* = 1173
Age					
	18-29	16	14	31	30
	30-49	30	30	41	43
	50-64	27	27	19	18
	65+	27	28	10	9
Gender					
	Male	49	49	52	49
	Female	51	51	48	51
Education					
	No education^1^	-	-	-	-
	Primary school	17	37	78	55
	Secondary or vocational school	51	43	9	31
	Bachelor	19	6	10	13
	Master and above	14	15	3	1
Income					
	0-17,999€	20	—	—	—
	18,000-29,999€	27	—	—	—
	30,000-47,999€	27	—	—	—
	48,000-59,999€	11	—	—	—
	more than 60,000€	16	—	—	—
	0-15,000€	—	45	—	—
	15,000-26,000€	—	29	—	—
	26,000-35,000€	—	13	—	—
	35,000-70,000€	—	10	—	—
	more than 70,000€	—	3	—	—
	0-60,000INR	—	—	22	—
	60,001-90,000INR	—	—	26	—
	90,001INR-120,000INR	—	—	20	—
	120,001-240,000INR	—	—	20	—
	240,001-600,000INR	—	—	11	—
	600,001-1200,000INR	—	—	2	—
	more than 1200,001INR	—	—	0	—
	0-212,400ZAR	—	—	—	31
	212,401-356,400ZAR	—	—	—	37
	356,401-500,400ZAR	—	—	—	18
	500,401-740,400ZAR	—	—	—	9
	more than 740,401ZAR	—	—	—	5

*Note.* Target values reflect external population margins used for stratification/weighting. Percentages are rounded to whole numbers; totals may not sum to exactly 100% due to rounding. ^1^No education was not quota-tracked; retained in the questionnaire and adjusted via weighting. “–” indicates no explicit target was specified for this category.

**Table 4:** Germany (n = 1003): sample composition vs. targets.

Variable	Category	Target %	Sample % (unweighted)	Sample % (weighted)	Δ (weighted - Target)
Age					
	18-29	16	15	14	-2
	30-49	30	32	31	1
	50-64	27	24	26	-1
	65+	27	29	29	2
Gender					
	Male	49	51	49	0
	Female	51	49	51	0
Education					
	No education^1^	-	1	1	-
	Primary school	17	5	5	-12
	Secondary or vocational school	51	64	62	11
	Bachelor	19	16	19	0
	Master and above	14	14	14	0
Income					
	0-17,999€	20	20	20	0
	18,000-29,999€	27	24	27	0
	30,000-47,999€	27	28	26	-1
	48,000-59,999€	11	11	11	0
	more than 60,000€	16	17	16	0

*Note.* Target values reflect external population margins used for stratification/weighting. Percentages are rounded to whole numbers; totals may not sum to exactly 100% due to rounding. ^1^No education was not quota-tracked; retained in the questionnaire and adjusted via weighting. “–” indicates no explicit target was specified for this category.

**Table 5:** Italy (n = 1002): sample composition vs. targets.

Variable	Category	Target %	Sample % (unweighted)	Sample % (weighted)	Δ (weighted - Target)
Age					
	18-29	14	11	13	-1
	30-49	30	29	28	-2
	50-64	27	29	28	1
	65+	28	31	30	2
Gender					
	Male	49	49	49	0
	Female	51	51	51	0
Education					
	No education^1^	-	1	1	-
	Primary school	37	31	36	-1
	Secondary or vocational school	43	46	43	0
	Bachelor	6	6	6	0
	Master and above	15	15	15	0
Income					
	0-15,000€	45	41	45	0
	15,000-26,000€	29	31	29	0
	26,000-35,000€	13	14	13	0
	35,000-70,000€	10	11	10	0
	more than 70,000€	3	3	3	0

*Note.* Target values reflect external population margins used for stratification/weighting. Percentages are rounded to whole numbers; totals may not sum to exactly 100% due to rounding. ^1^No education was not quota-tracked; retained in the questionnaire and adjusted via weighting. “–” indicates no explicit target was specified for this category.

**Table 6:** India (n = 1038): sample composition vs. targets.

Variable	Category	Target %	Sample % (unweighted)	Sample % (weighted)	Δ (weighted - Target)
Age					
	18-29	31	31	30	-1
	30-49	41	41	40	-1
	50-64	19	19	20	1
	65+	10	9	10	0
Gender					
	Male	52	52	52	0
	Female	48	48	48	0
Education					
	No education^1^	-	2	2	-
	Primary school	78	74	75	-3
	Secondary or vocational school	9	10	9	0
	Bachelor	10	10	10	0
	Master and above	3	4	3	0
Income					
	0-60,000INR	22	24	22	0
	60,001-90,000INR	26	24	25	-1
	90,001INR-120,000INR	20	20	20	0
	120,001-240,000INR	20	19	19	-1
	240,001-600,000INR	11	12	11	0
	600,001-1200,000INR	2	2	2	0
	more than 1200,001INR	0	0	0	0

*Note.* Percentages are rounded to whole numbers; totals may not sum to exactly 100% due to rounding. ^1^No education was not quota-tracked; retained in the questionnaire and adjusted via weighting. No explicit target was specified for this category.

**Table 7:** South Africa (n = 1173): sample composition vs. targets.

Variable	Category	Target %	Sample % (unweighted)	Sample % (weighted)	Δ (weighted - Target)
Age					
	18-29	30	26	28	-2
	30-49	43	49	44	1
	50-64	18	20	19	1
	65+	9	6	9	0
Gender					
	Male	49	47	48	-1
	Female	51	53	52	1
Education					
	No education^1^	-	2	2	-
	Primary school	55	54	53	-2
	Secondary or vocational school	31	31	31	0
	Bachelor	13	12	13	0
	Master and above	1	1	1	0
Income					
	0-212,400ZAR	31	29	31	0
	212,401-356,400ZAR	37	37	37	0
	356,401-500,400ZAR	18	19	18	0
	500,401-740,400ZAR	9	9	9	0
	more than 740,401ZAR	5	5	5	0

*Note.* Percentages are rounded to whole numbers; totals may not sum to exactly 100% due to rounding. ^1^No education was not quota-tracked; retained in the questionnaire and adjusted via weighting. No explicit target was specified for this category.

Values are rounded to one decimal place; columns may not sum to 100% due to rounding.

**Table 8:** Furniture product types and number of units in the respondents' households (unweighted % with weighted % in brackets).

Furniture products	Units	Germany	Italy	India	South Africa
Single seater sofa	None	20.9(20.9)	60.9(61.0)	20.7(20.8)	16.0(15.1)
	1-2	63.1(63.5)	31.6(31.4)	48.0(47.9)	59.9(60.2)
	3-4	10.9(10.6)	5.0(5.0)	16.7(16.6)	17.7(18.3)
	5-6	2.3(2.3)	1.5(1.6)	6.6(6.7)	3.9(4.1)
	7-10	1.7(1.6)	0.5(0.5)	5.3(5.3)	1.5(1.6)
	11-15	0.7(0.7)	0.3(0.3)	1.8(1.9)	0.3(0.3)
	16-20	0.2(0.2)	0.0(0.0)	0.2(0.2)	0.3(0.3)
	More than 20	0.2(0.2)	0.2(0.2)	0.7(0.6)	0.3(0.2)
Two-seater sofa	None	45.5(45.9)	44.8(45.2)	32.0(32.3)	20.7(20.2)
	1-2	50.0(49.9)	52.7(52.2)	38.1(37.7)	64.8(64.6)
	3-4	3.0(2.8)	1.8(1.9)	19.0(18.9)	11.1(11.6)
	5-6	0.7(0.7)	0.3(0.3)	5.3(5.2)	2.6(2.6)
	7-10	0.4(0.4)	0.2(0.2)	4.2(4.4)	0.7(0.8)
	11-15	0.2(0.2)	0.1(0.1)	1.3(1.4)	0.1(0.1)
	16-20	0.2(0.2)	0.0(0.0)	0.0(0.0)	0.0(0.0)
	More than 20	0.0(0.0)	0.1(0.1)	0.1(0.1)	0.1(0.1)
Three-seater sofa	None	48.3(48.6)	46.6(47.3)	39.4(39.2)	40.0(39.7)
	1-2	48.1(47.9)	49.0(48.0)	36.4(36.3)	51.6(51.7)
	3-4	2.4(2.3)	3.9(4.2)	12.3(12.5)	6.7(6.8)
	5-6	0.8(0.8)	0.3(0.3)	6.4(6.3)	1.4(1.4)
	7-10	0.4(0.4)	0.0(0.0)	4.0(4.1)	0.3(0.3)
	11-15	0.1(0.1)	0.2(0.2)	1.3(1.5)	0.1(0.1)
	16-20	0.0(0.0)	0.0(0.0)	0.0(0.0)	0.0(0.0)
	More than 20	0.0(0.0)	0.0(0.0)	0.1(0.1)	0.0(0.0)
4+ seater sofa	None	68.8(69.3)	81.3(81.4)	49.0(48.9)	65.8(65.8)
	1-2	28.7(28.4)	16.5(16.4)	26.8(26.6)	26.2(26.1)
	3-4	1.4(1.3)	1.8(1.9)	13.4(13.4)	6.1(6.1)
	5-6	0.6(0.5)	0.1(0.1)	5.7(5.8)	1.6(1.8)
	7-10	0.3(0.3)	0.3(0.3)	3.9(3.9)	0.2(0.1)
	11-15	0.2(0.2)	0.0(0.0)	1.2(1.2)	0.2(0.2)
	16-20	0.0(0.0)	0.0(0.0)	0.1(0.1)	0.0(0.0)
	More than 20	0.0(0.0)	0.0(0.0)	0.0(0.0)	0.0(0.0)
Single bed	None	38.9(39.0)	31.7(32.0)	14.4(14.4)	28.6(27.7)
	1-2	51.3(51.6)	60.1(59.7)	57.9(57.7)	58.6(59.0)
	3-4	8.2(7.8)	7.2(7.3)	16.8(16.8)	10.7(11.0)
	5-6	1.0(0.9)	0.5(0.5)	6.4(6.3)	1.8(2.0)
	7-10	0.5(0.5)	0.4(0.4)	2.8(2.9)	0.3(0.3)
	11-15	0.0(0.0)	0.1(0.1)	1.6(1.8)	0.0(0.0)
	16-20	0.1(0.1)	0.0(0.0)	0.1(0.1)	0.0(0.0)
	More than 20	0.0(0.0)	0.0(0.0)	0.1(0.1)	0.0(0.0)
Double bed	None	20.4(20.7)	5.4(5.8)	19.8(20.0)	11.8(11.4)
	1-2	74.1(74.0)	90.6(90.0)	49.6(49.3)	63.8(62.2)
	3-4	4.2(4.1)	3.3(3.4)	19.9(20.0)	21.7(23.3)
	5-6	1.0(0.9)	0.5(0.6)	6.0(5.9)	2.4(2.8)
	7-10	0.2(0.2)	0.1(0.1)	2.8(2.9)	0.2(0.2)
	11-15	0.1(0.1)	0.1(0.1)	1.7(1.9)	0.2(0.2)
	16-20	0.0(0.0)	0.0(0.0)	0.0(0.0)	0.0(0.0)
	More than 20	0.0(0.0)	0.0(0.0)	0.1(0.1)	0.0(0.0)
Chairs	None	1.8(1.8)	1.3(1.4)	10.1(10.4)	7.2(6.8)
	1-2	10.4(10.5)	4.1(4.4)	29.4(29.5)	25.7(25.5)
	3-4	23.6(23.7)	13.7(14.4)	27.5(26.7)	27.6(28.1)
	5-6	27.5(27.5)	27.7(28.0)	20.8(21.0)	22.8(22.7)
	7-10	25.3(25.5)	33.6(32.8)	9.7(9.9)	12.0(12.2)
	11-15	8.9(8.7)	13.9(13.4)	1.9(1.8)	3.2(3.3)
	16-20	1.5(1.5)	3.9(3.7)	0.5(0.5)	0.8(0.8)
	More than 20	1.0(0.9)	1.8(1.8)	0.1(0.1)	0.7(0.6)
Tables	None	0.5(0.5)	0.8(0.9)	10.5(10.5)	6.5(6.2)
	1-2	51.4(51.6)	75.1(75.7)	52.0(52.0)	58.7(58.4)
	3-4	36.6(36.7)	19.8(19.2)	24.6(24.5)	26.0(26.3)
	5-6	8.5(8.3)	3.4(3.4)	8.2(8.0)	6.6(6.9)
	7-10	2.1(2.0)	0.8(0.7)	3.0(3.1)	2.0(2.1)
	11-15	0.7(0.7)	0.1(0.1)	1.5(1.7)	0.1(0.1)
	16-20	0.2(0.2)	0.0(0.0)	0.1(0.1)	0.0(0.0)
	More than 20	0.0(0.0)	0.0(0.0)	0.1(0.1)	0.0(0.0)

*Note.* Entries are unweighted percentages. Values are rounded to one decimal place; columns may not sum to 100% due to rounding.

**Table 9:** Electrical/electronic product types and number of units in the respondents' households (Unweighted % with Weighted % in brackets).

Electronic/electrical products	Units	Country			
		Germany	Italy	India	South Africa
		*n* = 1003	*n* = 1002	*n* = 1038	*n* = 1173
Air conditioners					
	None	76.5(77.0)	38.4(39.0)	34.9(34.6)	39.4(39.2)
	1	15.4(15.2)	30.4(30.4)	36.0(36.4)	38.1(37.6)
	2	3.5(3.4)	17.1(16.9)	15.1(14.9)	13.0(13.1)
	3	2.0(1.9)	8.1(7.9)	6.3(6.4)	5.2(5.8)
	4	0.8(0.8)	3.7(3.5)	3.0(2.9)	2.3(2.3)
	5	0.3(0.3)	1.3(1.3)	2.1(2.3)	0.6(0.7)
	6	0.1(0.1)	0.5(0.5)	1.3(1.3)	0.7(0.7)
	More than 7	1.5(1.5)	0.5(0.5)	1.3(1.2)	0.8(0.7)
Laundry machines					
	None	3.2(3.1)	1.7(1.8)	27.3(26.9)	11.5(12.1)
	1	89.7(90.0)	92.3(92.3)	46.1(45.9)	71.1(69.7)
	2	5.5(5.4)	5.3(5.2)	16.5(16.7)	12.3(12.9)
	3	0.9(0.8)	0.4(0.4)	4.7(4.8)	2.8(2.8)
	4	0.2(0.2)	0.1(0.1)	1.9(2.0)	1.6(1.8)
	5	0.3(0.3)	0.1(0.1)	1.8(1.9)	0.3(0.3)
	6	0.0(0.0)	0.1(0.1)	1.4(1.5)	0.1(0.1)
	More than 7	0.2(0.2)	0.0(0.0)	0.3(0.3)	0.3(0.3)
Dishwashers					
	None	22.2(22.6)	44.2(46.0)	42.6(42.2)	41.3(41.3)
	1	73.7(73.5)	53.5(51.7)	34.1(34.4)	45.8(45.1)
	2	2.7(2.6)	1.5(1.5)	12.3(12.0)	8.7(8.9)
	3	0.6(0.6)	0.3(0.3)	4.3(4.4)	2.8(3.1)
	4	0.3(0.2)	0.3(0.3)	3.4(3.5)	0.8(0.9)
	5	0.5(0.5)	0.2(0.2)	2.1(2.3)	0.4(0.5)
	6	0.0(0.0)	0.0(0.0)	0.6(0.6)	0.1(0.1)
	More than 7	0.0(0.0)	0.0(0.0)	0.6(0.6)	0.1(0.1)
Refrigerators					
	None	0.6(0.6)	1.0(1.1)	11.8(11.8)	3.2(3.3)
	1	74.6(74.9)	83.9(84.0)	60.3(60.1)	61.6(59.8)
	2	20.0(19.7)	12.6(12.5)	15.0(15.0)	27.5(28.2)
	3	3.6(3.7)	1.8(1.8)	5.4(5.4)	5.8(6.5)
	4	0.5(0.5)	0.4(0.4)	2.7(2.8)	1.1(1.2)
	5	0.3(0.2)	0.2(0.2)	2.9(3.0)	0.4(0.5)
	6	0.1(0.1)	0.0(0.0)	1.1(1.0)	0.3(0.3)
	More than 7	0.3(0.3)	0.1(0.1)	0.8(0.8)	0.2(0.2)
Computers and laptops					
	None	3.1(3.1)	11.8(12.8)	23.9(23.9)	8.9(9.5)
	1	36.5(37.2)	47.5(47.4)	40.8(40.8)	39.4(38.3)
	2	35.4(35.3)	26.9(26.3)	19.4(19.1)	29.2(29.5)
	3	15.5(15.1)	8.4(8.1)	7.1(7.4)	14.2(14.3)
	4	5.8(5.6)	3.9(4.0)	4.1(4.2)	5.5(5.5)
	5	2.4(2.3)	1.1(1.1)	2.3(2.4)	1.4(1.4)
	6	0.7(0.7)	0.4(0.4)	1.2(1.2)	1.1(1.1)
	More than 7	0.7(0.6)	0.0(0.0)	1.2(1.1)	0.3(0.3)
TVs					
	None	4.5(4.6)	1.8(1.9)	10.3(10.5)	5.1(5.1)
	1	45.4(46.2)	31.0(31.8)	52.1(51.9)	49.3(48.9)
	2	31.9(31.5)	36.8(36.9)	22.4(22.0)	29.7(29.8)
	3	13.0(12.8)	22.1(21.2)	6.9(7.1)	11.0(10.9)
	4	3.5(3.3)	6.5(6.4)	3.7(3.7)	3.6(3.8)
	5	1.1(1.0)	1.6(1.6)	2.3(2.4)	0.9(1.0)
	6	0.5(0.4)	0.1(0.1)	1.3(1.4)	0.3(0.3)
	More than 7	0.2(0.2)	0.1(0.1)	1.0(1.0)	0.2(0.2)
Phones (all types of phones)					
	None	2.8(2.8)	2.1(2.1)	6.0(6.3)	2.4(2.4)
	1	25.4(25.7)	14.5(14.7)	25.7(25.5)	18.1(17.5)
	2	28.8(29.0)	30.2(30.7)	19.3(19.3)	19.9(20.0)
	3	21.7(21.8)	26.3(25.8)	20.8(20.7)	23.8(23.2)
	4	11.3(11.1)	15.1(14.9)	14.4(14.5)	16.8(16.8)
	5	5.5(5.3)	6.7(6.5)	8.6(8.7)	7.9(8.6)
	6	2.5(2.5)	3.6(3.7)	2.7(2.6)	5.2(5.6)
	More than 7	2.0(1.9)	1.5(1.7)	2.6(2.5)	5.9(6.1)

*Note.* Entries are weighted percentages. Values are rounded to one decimal place; columns may not sum to 100% due to rounding.

**Table 10:** How much do you engage with the following sustainable consumption practices within the circular economy? (unweighted % with weighted % in brackets)

		Never	Rarely	Sometimes	Always		
Germany	I repair/maintain my belongings or have them repaired/maintained.	1.7(1.7)	9.4(9.4)	58.0(57.7)	30.9(31.3)		
	I reuse items by extending their lifespan or purchasing second-hand products.	1.5(1.4)	9.4(9.4)	52.4(52.1)	36.7(37.0)		
	I recycle the products after using them	1.5(1.4)	8.8(8.6)	29.9(29.6)	59.8(60.3)		
	I purchase refurbished products	12.0(11.9)	30.3(30.7)	48.7(48.5)	9.1(9.0)		
	I prefer sharing over ownership of products	25.0(25.2)	32.5(32.4)	32.1(32.4)	10.4(10.1)		
Italy	I repair/maintain my belongings or have them repaired/maintained.	2.7(2.9)	9.1(9.3)	50.8(51.2)	37.4(36.6)		
	I reuse items by extending their lifespan or purchasing second-hand products.	4.9(4.9)	12.7(13.0)	50.9(51.1)	31.5(31.0)		
	I recycle the products after using them	4.7(4.7)	11.1(11.1)	43.2(43.4)	41.0(40.8)		
	I purchase refurbished products	19.2(19.2)	29.0(29.2)	42.9(42.5)	8.9(9.1)		
	I prefer sharing over ownership of products	28.1(28.0)	29.5(29.2)	33.3(33.6)	9.0(9.2)		
India	I repair/maintain my belongings or have them repaired/maintained.	3.3(3.3)	5.3(5.1)	34.1(34.4)	57.3(57.2)		
	I reuse items by extending their lifespan or purchasing second-hand products.	4.6(4.7)	8.8(8.8)	51.4(51.2)	35.2(35.2)		
	I recycle the products after using them	6.3(6.4)	12.2(12.3)	39.3(39.3)	42.2(42.0)		
	I purchase refurbished products	12.0(12.0)	14.3(13.7)	46.0(46.7)	27.7(27.5)		
	I prefer sharing over ownership of products	10.9(10.8)	12.7(12.5)	40.3(40.5)	36.1(36.2)		
South Africa	I repair/maintain my belongings or have them repaired/maintained.	1.1(1.2)	4.0(4.4)	43.1(43.3)	51.8(51.2)		
	I reuse items by extending their lifespan or purchasing second-hand products.	1.5(1.6)	7.2(7.7)	50.6(50.4)	40.8(40.3)		
	I recycle the products after using them	1.0(1.0)	9.9(10.3)	46.6(46.6)	42.5(42.1)		
	I purchase refurbished products	3.5(3.8)	15.4(16.8)	52.7(51.9)	28.4(27.5)		
	I prefer sharing over ownership of products	7.0(7.3)	20.8(21.3)	44.6(44.6)	27.6(26.9)		

Variable	Question	Question details	Germany	Italy	India	South Africa	Cronbach's Alpha
Intention	How likely do you think you are to adjust your habits to contribute to circular economy for sustainable and environmental conscious lifestyle?	I will refrain from purchasing and owning many things because I want to reduce my consumption.	3.25	3.42	3.10	3.41	0.87
		I will donate or sell items that are no longer needed to extend product life and reduce waste.	3.75	3.70	3.36	3.81	
		I will purchase second-hand items to reduce the need for buying new ones.	3.20	3.18	3.46	3.56	
		I will participate in product take-back or recycling programs instead of disposing off them.	3.93	3.47	3.49	3.79	
		I will purchase energy-efficient electronic or electric products to reduce climate emissions.	3.93	3.90	3.55	3.87	
		I will buy quality products that may cost more but last longer.	3.75	3.77	3.70	3.95	
		I will purchase products that can be refilled/reused.	3.94	3.94	3.74	4.04	
Social influence	To what extent do recommendations from groups listed below influence your decisions to purchase circular economy products?	Family, friends and neighbours	3.36	3.25	4.04	3.89	0.85
		Peers	3.09	3.05	3.63	3.67	
		Educational institutes	2.93	3.02	3.78	3.79	
		Social media	2.92	3.00	3.87	3.98	
		Environmental organization working with circular economy	3.29	3.44	4.04	4.08	
Barriers	What do you perceive as the most significant barriers to adopting circular economy practices in your daily life?	I do not know enough about circular economy practices and products.	3.18	3.36	3.91	3.34	0.90
		I do not buy circular economy products because of higher prices.	2.95	3.18	3.63	3.12	
		There are no incentives or rewards for circular economy behaviour.	3.35	3.51	3.69	3.15	
		There is a limited availability of circular products in the market.	3.44	3.54	3.74	3.27	
		I lack trust in the quality of circular products available in the market.	2.89	2.95	3.71	3.07	
		I lack trust in the quality of repair services available in the market for circular products.	2.87	2.91	3.72	3.12	
		I doubt that buying circular economy products will contribute to environmental sustainability.	2.71	2.73	3.84	2.92	
		I believe that lower value is associated with purchasing repaired, refurbished, or recycled goods in the society.	2.93	2.87	3.87	3.47	
		I believe societal pressure to own the latest electronic gadgets or furniture hinders buying circular products.	3.28	3.63	3.80	3.51	
		I think it is easier to buy new products compared to circular economy products.	3.37	3.35	3.65	3.33	
		I do not know how I can contribute in circular economy.	2.63	2.89	3.49	3.00	
		I am concerned that circular economy practices could lead to job losses.	2.50	2.79	3.59	3.07	
Motivations	What do you perceive as the important motivations for adopting circular economy practices in your daily life.	I feel a personal responsibility to engage in circular economy for the better environment.	3.25	3.42	3.10	3.41	0.84
		I purchase circular economy products due to my values and principles for a sustainable environment.	3.60	3.69	3.89	4.02	
		People's positive attitude towards circular practices encourages my inclination towards circular consumption.	3.45	3.52	3.95	3.98	
		Engaging in the circular economy gives me a sense of being part of a community or society actively working to make the earth a better planet.	3.45	3.60	3.98	4.02	

Percentages are rounded to whole numbers; totals may not sum to exactly 100% due to rounding.

## Limitations

Several limitations should be considered when using this dataset. First, although stratified sampling and post-stratification weights were applied to approximate national populations, the samples may still deviate from true population characteristics due to coverage limitations and unobserved sources of bias. The data should therefore be interpreted as broadly representative rather than perfectly reflective of national populations.

Second, the data are based on self-reported responses, which may be subject to recall bias, social desirability bias, or misreporting of behaviours and attitudes. As such, caution is advised when interpreting absolute levels of engagement in CE practices.

Third, the dataset captures a single time point (March–April 2024), which precludes analysis of behavioural dynamics or causal relationships over time. Longitudinal follow-up surveys would be required to assess how consumer awareness, motivations, and adoption of CE practices evolve in response to policies, market changes, or societal developments.

Fourth, the survey did not rely on previously validated scales for measuring attitudes, motivations, and barriers. Instead, items were developed specifically for this study and tailored to the context of circular economy practices. While this approach ensured relevance to the research objectives, it limits direct comparability with studies that employ standardized measurement instruments. Internal consistency was assessed for grouped multi-item measures using Cronbach’s alpha. However, the measures should not be interpreted as fully validated psychometric scales. Whereas the formal assessment of convergent validity would require dedicated validation analyses beyond the scope of this Data in Brief article.

Finally, while the survey covers four countries across Europe, Asia, and Africa, findings should not be generalized to other regions without careful consideration of contextual differences in cultural norms, economic conditions, and CE infrastructure.

## Ethics Statement

This study complied with established research ethics guidelines. Ethical approval for the survey was obtained from Norwegian Agency for Shared Services in Education and Research (SIKT: reference number 366071). Participation was voluntary, and respondents could withdraw at any time. The survey was designed to ensure anonymity: no personal identifiers (e.g., names, addresses, contact details) were collected, and only anonymized response data were provided by the external survey provider (Efficience3), which handled recruitment and initial processing. The study complied with GDPR requirements for personal data processing. The anonymized dataset is openly available for research and practical applications related to circular economy and sustainable consumption.

## CRediT Author Statement

**Altamash Bashir:** Conceptualization, Methodology, Formal analysis, Investigation, Writing -- original draft; **Lena Senft:** Conceptualization, Methodology, Validation, Formal analysis, Data Curation, Writing -- review & editing, Visualization; **Christian A. Klöckner:** Resources, Writing -- review & editing, Supervision, Project administration, Funding acquisition. Bashir and Senft share first authorship.

## Data Availability

Zenodo.orgSurvey data on consumer circular economy behaviours: Focus on repair, reuse, recycling, refurbishment, and sharing in electronics and furniture across Germany, Italy, India and South Africa (Original data)
